# PSMA expression and PSMA PET/CT imaging in metastatic soft tissue sarcoma patients, results of a prospective study

**DOI:** 10.1007/s00259-025-07224-z

**Published:** 2025-03-27

**Authors:** F. Kleiburg, T. van der Hulle, H. Gelderblom, M. Slingerland, F. M. Speetjens, L. J.A.C. Hawinkels, P. Dibbets-Schneider, F. H.P. van Velden, M. Pool, S. W. Lam, J. V.M.G. Bovée, L. Heijmen, L. F. de Geus-Oei

**Affiliations:** 1 Biomedical Photonic Imaging Group, University of Twente, Enschede, The Netherlands; 2 Department of Radiology, Section of Nuclear Medicine, Leiden University Medical Center, Leiden, The Netherlands; 3 Department of Medical Oncology, Leiden University Medical Center, Leiden, The Netherlands; 4 Department of Gastroenterology, Leiden University Medical Center, Leiden, The Netherlands; 5 Department of Clinical Pharmacy and Toxicology, Leiden University Medical Center, Leiden, The Netherlands; 6 Department of Pathology, Leiden University Medical Center, Leiden, The Netherlands; 7 Department of Radiation Science and Technology, Technical University of Delft, Delft, The Netherlands

**Keywords:** soft tissue sarcoma, prostate-specific membrane antigen, PSMA, PSMA PET/CT, molecular imaging, theranostics

## Abstract

**Purpose:**

Prostate-specific membrane antigen (PSMA) expression has been observed in a subset of soft tissue sarcomas, mainly in the neovascular endothelial cells. This feasibility study aimed to evaluate PSMA expression and PSMA PET/CT imaging in metastatic soft tissue sarcoma, providing important insights for potential future exploration of PSMA-targeted radioligand therapy.

**Methods:**

This prospective single-center study included adult patients with metastatic soft tissue sarcoma, with measurable disease (lesion diameter > 1 cm), available biopsy/resection material, ECOG/WHO performance status of 0–2 and either no prior systemic treatment, progressive disease during/after treatment, or stable disease/partial response with the last dose > 8 weeks prior. Immunohistochemical PSMA staining was performed on previously obtained biopsy or resection material. In case of high PSMA expression, a [^18^F]-JK-PSMA-7 PET/CT scan evaluated tracer uptake, with adequate uptake defined as SUV_max_ > 8.

**Results:**

Of 25 included patients, 11 (44%) had high PSMA expression: 4/11 leiomyosarcomas, 3/4 dedifferentiated liposarcomas, 2/5 undifferentiated pleomorphic sarcomas, 1/2 myxofibrosarcomas and 1/1 malignant peripheral nerve sheath tumour. Five of 11 patients agreed to a [^18^F]-JK-PSMA-7 PET/CT, of which 3 had lesions that showed adequate tracer uptake (SUV_max_ 10.7–16.7). However, uptake across all metastatic lesions was highly heterogeneous (median SUV_max_ = 3.8; range 0.5–16.7), indicating that these patients are unlikely to benefit sufficiently from PSMA-targeted therapy. The study was therefore terminated prematurely.

**Conclusion:**

PSMA expression and PSMA tracer uptake in metastatic soft tissue sarcoma were highly heterogeneous. A deeper understanding of PSMA biology and improved patient selection criteria are essential for future application of PSMA-targeted radioligand therapy in this disease.

**Trial registration:**

: clinicaltrials.gov, NCT05522257. Registered 31-08-2022.

## Introduction

Soft tissue sarcomas represent a heterogeneous group of rare malignancies arising from mesenchymal tissues, with an incidence of approximately 5 cases per 100,000 individuals annually [1]. It can develop in many different anatomical locations and over 70 histological subtypes have been identified [2]. Due to its diverse and heterogeneous origin, it can manifest with a wide range of clinical presentations and variable responses to treatment, making the diagnosis and management of soft tissue sarcomas challenging. For metastatic soft tissue sarcomas, standard of care consists of cytotoxic chemotherapy, with doxorubicin being the first choice for the majority of soft tissue sarcoma subtypes [3]. However, approximately 14% of patients respond to doxorubicin treatment [3, 4], and the five-year survival rate for metastatic soft tissue sarcoma is only 17% [5]. This highlights the need to explore new effective treatment options to improve patient outcomes.

Prostate-specific membrane antigen (PSMA) is a type II transmembrane glycoprotein best known for its upregulated expression in the epithelium of prostate cancer cells, where it serves as an important target for molecular imaging (PSMA-targeted PET/CT scans [6]) and radioligand therapy (e.g. [^177^Lu]Lu-PSMA-617 and [^225^Ac]Ac-PSMA-617 [7, 8]). Interestingly, studies have demonstrated PSMA expression in the tumour-associated neovascular endothelial cells of various other malignancies, including soft tissue sarcomas [9]. This was first described in 2017 by Heitkötter et al., who analysed 779 sarcoma samples and reported strong neovascular PSMA expression in various sarcoma entities, such as pleomorphic rhabdomyosarcoma (40% of samples) and synovial sarcoma (38% of samples) [10]. Malignant soft tissue tumours showed evidently higher PSMA expression compared to tumours with benign and intermediate biological potential. Multiple case reports have confirmed PSMA tracer uptake in patients with soft tissue sarcoma, with uptake ranging from mild to intense in e.g. liposarcoma, undifferentiated pleomorphic sarcoma, angiosarcoma and leiomyosarcoma [11]. Notably, high PSMA tracer uptake has been observed particularly in aggressive histological subtypes and metastatic disease, with maximum standardised uptake values (SUV_max_) of up to 17 [12–14]. In one case, PSMA tracer uptake differentiated dedifferentiated liposarcoma from lipomatous regions [15], while in another case, tracer uptake increased with progression of metastatic leiomyosarcoma [13]. These findings suggest that PSMA expression, if present, becomes more pronounced in more advanced soft tissue sarcomas. Therefore, we hypothesised that effective PSMA-ligand binding may be achievable in selected patients with PSMA-positive metastatic soft tissue sarcoma, potentially enabling PSMA-targeted radioligand therapy (PSMA-RLT) in this patient population most in need of new treatment options. However, no prospective studies have been performed yet to investigate this potential.

The aim of this prospective feasibility study was to investigate immunohistochemical PSMA expression in metastatic soft tissue sarcomas and to evaluate tracer uptake on PSMA PET/CT imaging in patients with confirmed PSMA-expressing soft tissue sarcomas. This will provide important insights for potential future exploration of PSMA theranostics in soft tissue sarcomas.

## Materials and methods

### Study design

The study was a single-center, open-label, feasibility study in patients with metastatic soft tissue sarcomas, conducted at the Leiden University Medical Center (Leiden, The Netherlands). It was a non-randomized, non-blinded study to assess the level of PSMA expression in biopsy or resection material from soft tissue sarcomas and, in case of confirmed high PSMA expression, to assess the amount of tumoural PSMA-tracer binding on a [^18^F]-JK-PSMA-7 PET/CT scan (in short: PSMA PET/CT scan). This study was approved by the Medical Ethics Committee Leiden The Hague Delft and was registered on clinicaltrials.gov (NCT05522257, registry date 31-08-2022).

### Eligibility criteria

The inclusion criteria were: (1) diagnosis of metastatic (nodal or distant) soft tissue sarcoma; (2) age ≥ 18 years at the time of written informed consent; (3) recent (< 8 weeks) standard imaging (with CT or [^18^F]FDG PET/CT) with measurable disease (lesion diameter > 1 cm); (4) biopsy or resection available of the primary tumour and/or metastasis; (5) ECOG/WHO performance status of 0–2; (6) either no previous systemic therapy for advanced soft tissue sarcoma, or, previous systemic therapy for advanced soft tissue sarcoma with progression of disease during or after discontinuation of systemic therapy, or, previous systemic therapy for advanced soft tissue sarcoma with partial response or stable disease where the last dose of systemic therapy was given > 8 weeks before.

The exclusion criteria were: (1) women who were pregnant and/or lactating; (2) medical or psychiatric conditions that compromised the patient’s ability to give informed consent; (3) known hypersensitivity to drugs comparative to [^18^F]-JK-PSMA-7, any of their excipients or to any component of [^18^F]-JK-PSMA-7; (4) inability to undergo PET/CT scanning, e.g. claustrophobia, body weight higher than the weight limit of the scanner or inability to tolerate lying down for the duration of a PET/CT scan.

### Study procedures

In all eligible patients, immunohistochemical PSMA staining was performed on formalin-fixed paraffin-embedded slides of biopsy or resection material that was obtained as part of standard clinical practice. Immunohistochemistry was performed with the anti-PSMA antibody (clone: D7I8E) (Cell Signalling, Danvers, MA, USA) at a dilution of 1:40. The EnVision detection system was used and all steps were performed on the DAKO Omnis (Agilent Technologies, Santa Clara, CA, USA). Antigen retrieval was carried out with a low pH, followed by incubation with the primary antibody for 27.5 min with the addition of a rabbit linker (10 min incubation time). PSMA expression levels were assessed by one pathologist and categorised into no expression, low expression or high expression as defined by Heitkötter et al. [10]. High PSMA expression was defined as moderate staining intensity in > 5% of the neovasculature or the presence of any strong staining.

An intravenous injection with a fixed dose of 359 ± 36 MBq [^18^F]-JK-PSMA-7 was administered 90 min before the PET/CT scan was acquired [16]. Images were obtained using the Philips Vereos (Philips Healthcare, Best, The Netherlands) or the Omni Legend 32 cm PET/CT scanner (GE Healthcare, Chicago, Illinois, United States of America), located at the Leiden University Medical Center. The scan range was from crown to mid-thigh, or from crown to toe in case the primary tumour was located in the lower extremities, and acquisition was carried out in supine position. All PET images underwent iterative reconstruction, compliant with the EARL1 harmonization criteria to ensure comparable SUVs [17]. All reported lesions on standard imaging were assessed on PSMA PET/CT and a 3-dimensional volume of interest was inserted around each lesion with a diameter > 1 cm. Thereafter, SUV_max_ values were extracted to quantify PSMA tracer uptake. As PSMA uptake is generally underestimated in smaller lesions due to the partial-volume effect [18], no quantification was performed in lesions < 1 cm.

### Study objectives

The primary study objective was to determine the number of patients in which a SUV_max_ higher than 8 was reached. The cut-off of 8 was chosen to identify patients that might benefit from PSMA-RLT in the future. The *EANM procedure guidelines for radionuclide therapy with*^*177*^*Lu-labelled PSMA-ligands* state that there is no consensus yet on the definition of “adequate” uptake for therapy in prostate cancer patients, but suggest to use the definition from the LuPSMA trial: SUV_max, tumour_ > 1.5 * SUV_mean, liver_ [19, 20]. However, reference-organ variability between different PSMA tracers becomes an issue here. The LuPSMA trial used [^68^Ga]Ga-PSMA-11 PET/CT scans, on which healthy liver tissue has an average SUV_mean_ of 4.8, while the uptake in healthy liver tissue on [^18^F]-JK-PSMA-7 is more than twice as high [16, 21]. In order to still have a comparable threshold, we chose an absolute threshold of SUV_max_ > 8 (at least 1.5 * 4.8) to investigate which lesions might have adequate uptake for potential treatment. In this feasibility study we aimed to perform a PSMA PET/CT scan in 15 patients. The protocol stated that the study would be terminated early if a total of 5 PSMA PET/CT scans showed no adequate tracer uptake in all or the majority of metastases.

### Statistical analysis

As this was a feasibility study, study analyses were performed to derive preliminary results that may provide insights for future research. Descriptive statistics were used to describe the study outcomes. IBM SPSS Statistics (version 25 or higher) was used to derive these descriptive statistics.

## Results

### Patient characteristics

A total of 25 patients were included for immunohistochemical PSMA staining. Their characteristics are described in Table 1. Seven different histological soft tissue sarcoma entities were seen, of which leiomyosarcoma was the most common (44%). At diagnosis, 5 patients (20%) had metastatic disease and the majority had FNCLCC (Fédération Nationale des Centres de Lutte Contre le Cancer) grade 2 (48%). The median time from diagnosis to study inclusion was 18 months (range 0–143 months). Two patients had received systemic therapy before study inclusion; both had progressive disease during or after previous systemic therapy. The other patients had not received any systemic therapy.

**Table 1:** Patient characteristics (*n* = 25). *FNCLCC (Fédération Nationale des centres de lutte Contre Le Cancer) grade unknown in 8 patients

Characteristic	Value
Age in years, median (range)	68(33–84)
Sex, n (%) Male Female	15(60%) 10(40%)
FNCLCC grade at diagnosis, n (%)* 1 2 3	1(4%) 12(48%) 4(16%)
Histological type, n (%) Leiomyosarcoma Dedifferentiated liposarcoma Undifferentiated pleomorphic sarcoma Myxofibrosarcoma Malignant peripheral nerve sheath tumour Sclerosing epithelioid fibrosarcoma Dermatofibrosarcoma protuberans	11(44%) 5(20%) 4(16%) 2(8%) 1(4%) 1(4%) 1(4%)

### PSMA expression levels

High PSMA expression was seen in 11 patients (44%); 4/11 leiomyosarcomas, 2/5 dedifferentiated liposarcomas, 3/4 undifferentiated pleomorphic sarcomas, 1/2 myxofibrosarcoma and 1/1 malignant peripheral nerve sheath tumour (MPNST), see Table 2. Low PSMA expression was seen in 7 patients (28%); 4/11 leiomyosarcomas, 2/5 dedifferentiated liposarcomas and 1/2 myxofibrosarcomas. No PSMA expression was seen in 7 patients (28%); 3/11 leiomyosarcomas, 1/5 dedifferentiated liposarcomas, 1/4 undifferentiated pleomorphic sarcomas, 1/1 sclerosing epithelioid fibrosarcoma and 1/1 dermatofibrosarcoma protuberans. In four patients, both neovascular and cellular PSMA expression were observed; two leiomyosarcomas (one with high and one with low PSMA expression), one dedifferentiated liposarcoma (with low PSMA expression) and one MPNST (with high PSMA expression). The other patients showed only neovascular PSMA expression.

**Table 2:** Number of patients with high PSMA expression per histological subtype of soft tissue sarcoma

Histological subtype	High PSMA expression
Leiomyosarcoma	4/11(36%)
Dedifferentiated liposarcoma	2/5(40%)
Undifferentiated pleomorphic sarcoma	3/4(75%)
Myxofibrosarcoma	1/2(50%)
Malignant peripheral nerve sheath tumour	1/1(100%)
Sclerosing epithelioid fibrosarcoma	0/1(0%)
Dermatofibrosarcoma protuberans	0/1(0%)

### PSMA PET/CT scans

**Fig. 1:**
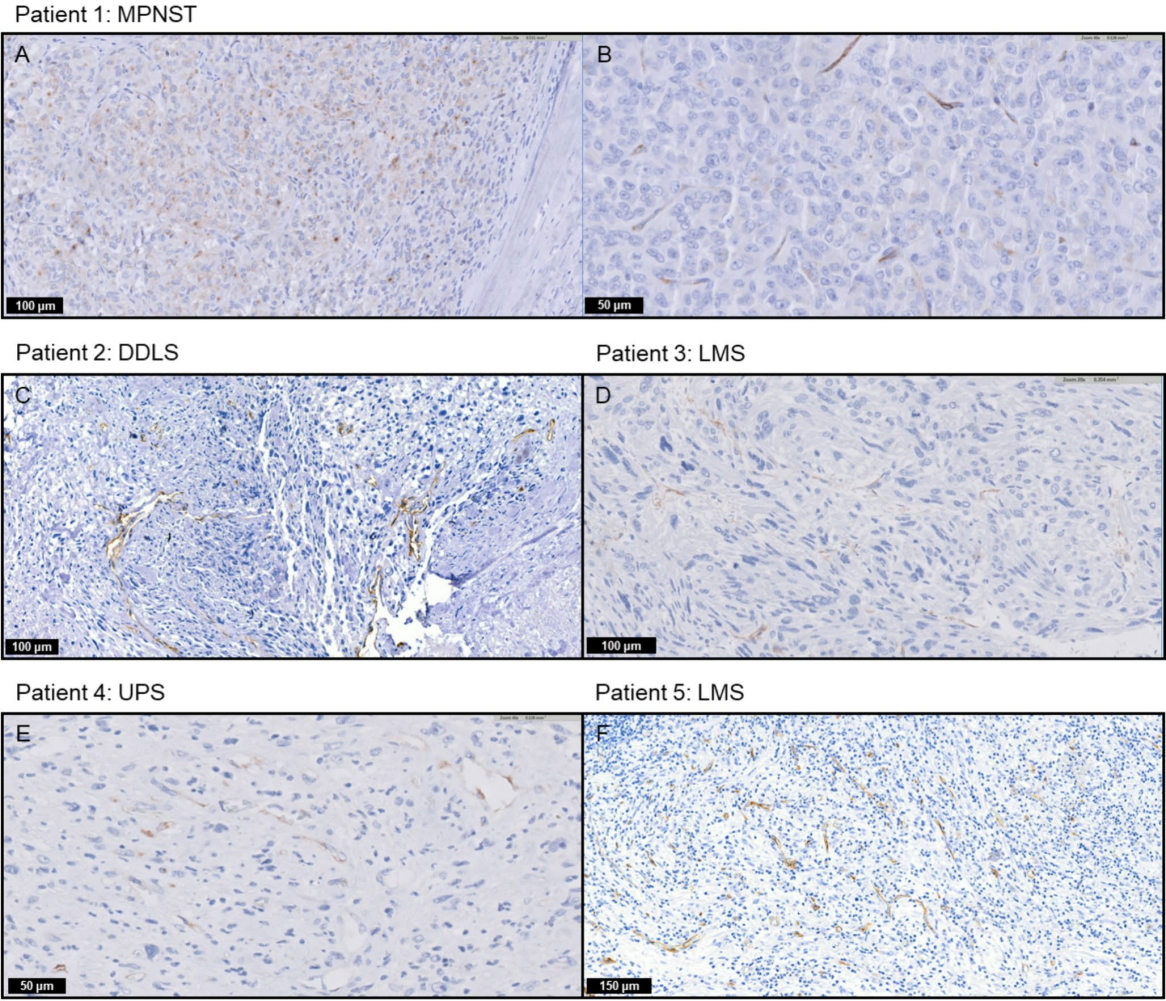
Immunohistochemical PSMA expression of the five patients that underwent PSMA PET/CT imaging. MPNST = malignant peripheral nerve sheath tumour, DDLS = dedifferentiated liposarcoma, LMS = leiomyosarcoma, UPS = undifferentiated pleomorphic sarcoma. Patient 1 had both cellular PSMA expression in approximately 30% of tumour cells (A) and focal neovascular PSMA expression (B). Patients 2–5 had neovascular PSMA expression in > 5% of blood vessels (C-F)

**Fig. 2:**
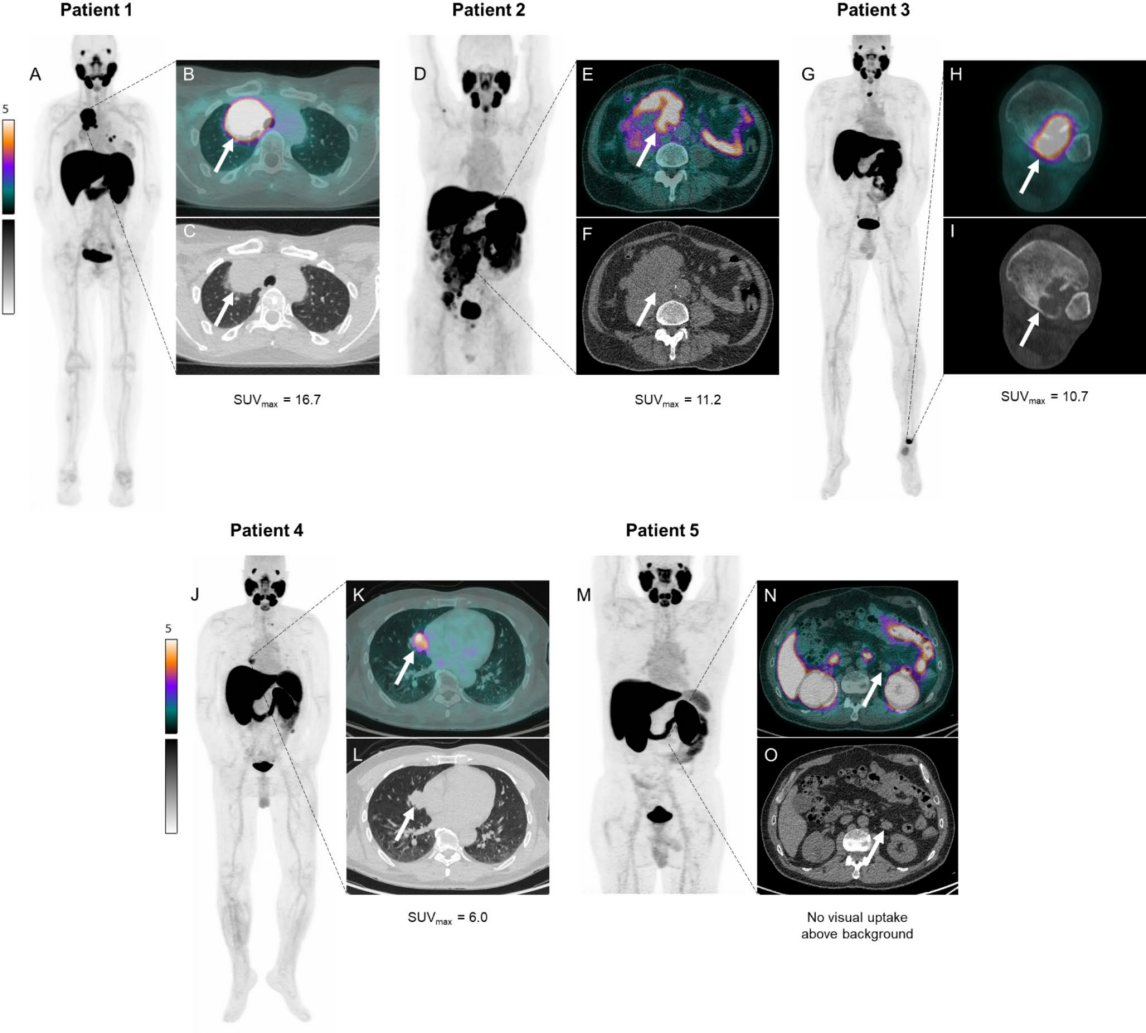
PSMA PET/CT images of the five scanned patients. White arrows indicate either the lesion with the highest SUV_max_ (patients 1 to 4, SUV_max_ = 6.0–16.7), or the largest lesions (patient 5, lesion caudal to left renal vein, no visual uptake above the background). A, D, G, J, M: maximum intensity projection (MIP) images. B, E, H, K, N: fused PET EARL1 and low-dose CT images. C, F, I, L, O: low-dose CT images

**Fig. 3:**
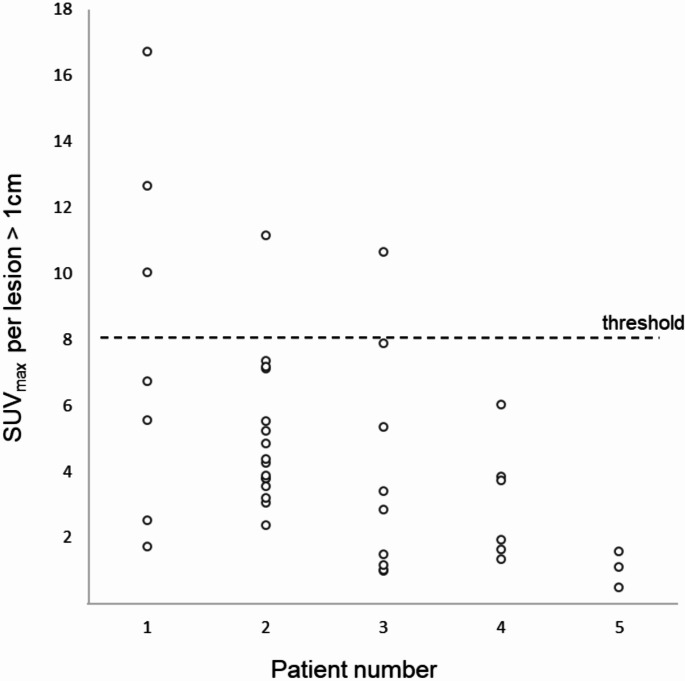
SUV_max_ values per lesion with a diameter > 1 cm. Five lesions had a SUV_max_ above the study threshold of 8. Immunohistochemical PSMA expression was done on resected lesions in all five patients, making these lesions unavailable for evaluation on PSMA PET/CT

Of the eleven patients with high PSMA expression, five agreed to undergo a PSMA PET/CT scan. The other six either did not want to participate (*n* = 3), or were not asked due to rapid clinical deterioration (*n* = 3). The immunohistochemical PSMA expression and PSMA PET/CT images of scanned patients can be seen in Figs. 1 and 2. Two patients were scanned with the Philips Vereos PET/CT scanner and three with the Omni Legend 32 cm PET/CT scanner. The patient characteristics, including details on PSMA expression and PSMA tracer uptake, are displayed in Table 3. In four patients immunohistochemical PSMA staining was performed on material from the primary tumour, and in one patient it was performed on resection material from a lung metastasis. In this patient, the primary tumour, which had been resected nine years earlier, was also assessed and showed no PSMA expression. An SUV_max_ > 8 was reached in three out of five patients; patient 1 had SUV_max_ = 16.7, patient 2 had SUV_max_ = 11.2 and patient 3 had SUV_max_ 10.7. Additionally, patient 4 showed moderate tracer uptake with an SUV_max_ = 6.0, and patient 5 showed no visual tracer uptake above the background. When analysing all lesions individually, high heterogeneity in tracer uptake was observed, see Fig. 3. Of 41 analysed lesions, 5 lesions (12%) had SUV_max_ > 8 and the median SUV_max_ of all lesions was 3.8 (range 0.5–16.7). Due to the heterogeneity, the included patients were not considered suitable for potential PSMA-targeted radioligand monotherapy and therefore, even though sufficient tracer uptake was seen in some lesions, the study was stopped early.

**Table 3:** Patient and disease characteristics, PSMA expression results and PSMA PET/CT results of the five scanned patients. None of these patients had received prior systemic treatment for soft tissue sarcoma

Patient number	Age	Sex	Initial diagnosis	Assessed biopsy/resection material	Interval from biopsy/resection to study inclusion	Localisation of PSMA expression	Lesions with SUV_max_ > 8 on PSMA PET/CT scan	Total number of lesions > 1 cm
1	33	F	Malignant peripheral nerve sheath tumour in right distal nervus tibialis, T1N0M0	Resection primary tumour	18 months	Cellular expression (~ 30%) and focal neovascular expression	Mediastinum: SUV_max_ = 16.7 Right hilum: SUV_max_ = 12.7 Subcarinal: SUV_max_ = 10.0	7
2	71	M	Dedifferentiated liposarcoma in right funiculus, T2N1M0	Biopsy primary tumour	2 months	Neovascular strong expression (~ 20%)	Retroperitoneal: SUV_max_ = 11.2	16
3	57	M	Leiomyosarcoma in left medial malleolus, T1N0M0	Resection lung metastasis left superior lobe*	6 months	Neovascular moderate expression (~ 20%)	Left tibia: SUV_max_ = 10.7	9
4	57	M	Undifferentiated pleomorphic sarcoma in right tibia, T1N0M0	Resection primary tumour	6 months	Neovascular moderate to strong expression (~ 40%)	None	6
5	61	M	Leiomyosarcoma in left adrenal gland, T2M0M0	Resection primary tumour	24 months	Neovascular moderate expression (~ 5%), a few vessels with strong expression	None	3

* 9 years after initial diagnosis. In this patient, immunohistochemical PSMA staining was also done on the resection specimen of the primary tumour (from 9 years earlier), which showed no PSMA expression

## Discussion

This study presents the first prospective data on PSMA expression and PSMA PET/CT imaging in patients with metastatic soft tissue sarcoma. PSMA expression was observed in a substantial proportion of patients, with variations across histological subtypes. PSMA PET/CT imaging showed adequate tracer binding in select lesions, however, within the patients in this study the tracer uptake was considered too heterogeneous for potential PSMA-RLT.

Of 25 included patients, representing seven different soft tissue sarcoma entities, 18 (72%) demonstrated PSMA-positive biopsy results, of which 11 (44%) showed high PSMA expression. These cases included leiomyosarcoma, dedifferentiated liposarcoma, undifferentiated pleomorphic sarcoma, myxofibrosarcoma and malignant peripheral nerve sheath tumour. Although our cohorts were small, the percentages of high PSMA expression in this study were higher than those reported by Heitkötter et al. [10], who applied the same definition. In their analysis of 779 tissue samples from a broad range of soft tissue and bone tumours, including 599 malignant tumours, they found PSMA expression in 20% of malignant tumours, with high PSMA expression in 7%. The difference in high PSMA expression rate was also evident within specific histological subtypes, for instance, 36% (4/11) of leiomyosarcomas in our study had high PSMA expression, compared to 11% (7/66) of leiomyosarcomas in the cohort of Heitkötter et al. Based on the association between PSMA expression and tumour aggressiveness, also within sarcomas [11], we hypothesise that the increased rates of high PSMA expression in this study stem from the inclusion criteria, which selected only patients who had developed metastatic soft tissue sarcoma. This finding is particularly relevant as these are the patients most in need of new treatment options. Interestingly, in one scanned patient a lung metastasis showed high PSMA expression, whereas the primary tumour resected nine years earlier had showed no PSMA expression, supporting the idea that PSMA expression can increase as the disease progresses. Furthermore, we observed a higher rate of cellular PSMA expression (4/25) compared to Heitkötter et al. (1/599), which may have implications for the feasibility of PSMA-RLT, as tumour cell expression could enhance treatment efficacy.

Five patients with high PSMA expression underwent PSMA PET/CT imaging, of which three met the criterium of SUV_max_ > 8. This indicates that adequate tracer binding may be achieved in over half of patients with high PSMA expression. However, PSMA uptake across all lesions was heterogeneous, with SUV_max_ values ranging from 0.5 to 16.7 (median 3.8) in lesions with a diameter of > 1 cm. Consequently, PSMA-RLT was not considered a feasible option for these patients and the study was stopped early. The decision to stop the study early was based on the pre-specified criteria and ethical considerations, as continuing with the methods and selection criteria of this study was unlikely to yield positive results given the observed heterogeneity. No research has yet investigated the underlying causes of heterogeneity in PSMA uptake within soft tissue sarcoma patients. We hypothesise that this variability may be due the evolution of different cancer subclones, each with varying levels of aggressiveness and PSMA expression. Other potential factors include differences in tumour microenvironment and in neovascularisation, which may promote PSMA expression in different ways, particularly as soft tissue sarcomas can occur in a wide variety of anatomical locations. In addition, two sarcoma patients treated with PSMA-RLT have been described in previous literature, both with metastatic leiomyosarcoma and heterogeneous PSMA uptake. In one case, the patient received a single dose of [^177^Lu]Lu-PSMA-617 (6.0 GBq), but treatment was discontinued due to poor radiotracer fixation in metastases observed on a whole body scan [13]. In the second case, two cycles of [^177^Lu]Lu-PSMA-I&T (dose unspecified) were combined with nivolumab, and post-treatment SPECT/CT showed marked uptake in lung metastases. However, treatment was stopped due to progressive disease [14]. These cases support the fact that there are still hurdles to overcome for potential PSMA-targeted treatment in sarcoma patients.

When considering PSMA-RLT in sarcoma patients, but also non-prostate tumours in general, several challenges must be addressed. Firstly, unlike prostate cancer where PSMA is expressed directly on tumour cells, PSMA is mostly restricted to neovascular endothelial cells in sarcoma and other non-prostate tumours [22]. Although the beta particle range of ^177^Lu-PSMA (approximately 1–2 mm) enables irradiation of surrounding tumour cells, the lower density of neovascular endothelial cells compared to tumour cells reduces available targets in the tumour. Secondly, PSMA expression and tracer uptake are often highly heterogeneous across lesions within the same patient, resulting in uneven radiation dose distribution. Thirdly, studies suggest that PSMA tracers in tumours with neovascular PSMA expression may exhibit faster washout, further diminishing the radiation dose. Fourthly, prior systemic treatments may have influenced PSMA expression, as some therapies have been reported to modulate PSMA levels. Lastly, the radiosensitivity of the targeted tumour has to be taken into account. Within soft tissue sarcoma cell lines there is a broad spectrum of radiosensitivities [23], meaning that some histological subtypes need higher absorbed radiation doses compared to others to achieve effective treatment. These factors collectively highlight the need for good understanding of PSMA biology and well-considered patient selection to reach potential effective PSMA-RLT.

Despite these challenges, some non-prostate cancers have shown successful response to ^177^Lu-PSMA treatment. For example, three glioblastoma patients achieved good tumour radiation doses, tumour shrinkage and improvement in performance status [24]. Similarly, multiple patients with adenoid cystic carcinoma achieved good clinical responses, such as pain reduction and symptom relief [24]. These results may be explained by the fact that glioblastoma is one of the most vascularised tumours, and that adenoid cystic carcinoma is known to be one of the few non-prostate tumours that expresses PSMA on tumour cells rather than its neovascular endothelial cells. These examples highlight the importance of patient selection strategies that consider tumour vascularisation and cellular PSMA expression patterns. However, the current literature is limited, and publication bias must be considered.

This study has several limitations. As this was an exploratory feasibility study with a small sample size, it was not possible to test for significant differences or associations, and no robust conclusions could be drawn. The diversity of the included soft tissue sarcoma subtypes added further complexity to comparisons, a common challenge in sarcoma research due to the wide variety of histological subtypes in combination with the rarity of tumours. Also, patients were selected based on immunohistochemical PSMA staining of biopsy or resection samples that were already previously obtained in a clinical setting. Sampling errors or long intervals between biopsy or resection and the development of metastases may have resulted in missed cases of high PSMA expression. In all scanned patients the immunohistochemical PSMA staining was performed on resected lesions, so the direct correlation between PSMA expression and PSMA tracer uptake could not be evaluated. Additionally, PSMA expression levels were assessed by one pathologist without a second reader, introducing potential variability, and patients were scanned at a single time point, so tracer retention time and absorbed radiation doses could not be assessed. Even though two different PET/CT scanners were used in this study, comparability of PET quantification was maximised by using EARL harmonisation. This standardisation helps to reduce potential variability in image quantification, thus ensuring reliable comparisons. No relevant differences in tumoural tracer accumulation are to be expected between [^18^F]-JK-PSMA-7, the tracer used in this study, and other commonly used PSMA tracers such as [^68^Ga]Ga-PSMA-11 [25].

For future studies investigating PSMA-targeted imaging and treatment in (soft tissue) sarcomas, it is crucial to focus on refining patient selection criteria to optimise identification of potentially eligible patients for PSMA-RLT without missing patients, while minimising unnecessary scans with no or insufficient tracer uptake and thus unnecessary patient burden and costs. For that, a broader cohort of patients has to be analysed. Also, limiting the time interval between tissue sampling and study inclusion may better capture relevant PSMA expression. To ensure consistency in immunohistochemical PSMA expression results, in future studies the assessment of inter-observer variability between pathologists, or automated quantification should be considered. To ensure consistent quantification of PSMA uptake across different scanners and studies, continued attention should be paid to PET harmonisation guidelines. A deeper understanding of PSMA biology is important for advancing the potential application of PSMA-RLT. Areas of interest include mechanisms that promote PSMA expression, both neovascular and cellular, factors underlying the observed heterogeneity in tracer uptake, and the influence of prior systemic treatments on PSMA expression. Additionally, the effect of neovascular PSMA expression on PSMA ligand binding and retention has to be analysed. Hopefully, the results of study NCT05420727, which aims to investigate the effect of [^177^Lu]Lu-PSMA-I&T in soft tissue sarcoma patients, will provide additional insights. In the future, perhaps exploring different theranostic targets simultaneously, such as PSMA and fibroblast activation protein inhibitors (FAPI), may lead to a more personalised theranostic approach for sarcoma patients.

## Conclusion

Although 44% of included patients exhibited high PSMA expression, and three out of five scanned patient had metastatic lesions with sufficient PSMA tracer uptake (SUV_max_ 10.7–16.7), the uptake across all lesions was deemed too heterogeneous to achieve adequate radiation doses. Consequently, this limits the potential for future effective radioligand treatment. To make PSMA-targeted radioligand treatment viable for patients with metastatic soft tissue sarcoma, a deeper understanding of PSMA biology and improved patient selection criteria are crucial. Larger trials are needed to further build on these preliminary results and explore potential strategies to improve patient selection and PSMA ligand binding. If current challenges can be overcome, PSMA theranostics may still hold promise for selected sarcoma patients.

## Data Availability

Not applicable.

## References

[1] Stiller CA, Trama A, Serraino D, Rossi S, Navarro C, Chirlaque MD, et al. Descriptive epidemiology of sarcomas in Europe: report from the RARECARE project. Eur J Cancer. 2013;49:684–95. 10.1016/j.ejca.2012.09.011. 23079473 10.1016/j.ejca.2012.09.011

[2] WHO Classification of Tumours Editorial Board. WHO Classification of tumours of soft tissue and bone. 5th ed. Lyon, France: IARC; 2020.

[3] Gronchi A, Miah AB, Dei Tos AP, Abecassis N, Bajpai J, Bauer S, et al. Soft tissue and visceral sarcomas: ESMO-EURACAN-GENTURIS Clinical Practice Guidelines for diagnosis, treatment and follow-up(). Ann Oncol. 2021;32:1348–65. 10.1016/j.annonc.2021.07.006. 34303806 10.1016/j.annonc.2021.07.006

[4] Judson I, Verweij J, Gelderblom H, Hartmann JT, Schoffski P, Blay JY, et al. Doxorubicin alone versus intensified doxorubicin plus ifosfamide for first-line treatment of advanced or metastatic soft-tissue sarcoma: a randomised controlled phase 3 trial. Lancet Oncol. 2014;15:415–23. 10.1016/S1470-2045(14)70063-4. 24618336 10.1016/S1470-2045(14)70063-4

[5] SEER Cancer Stat Facts. Soft Tissue Cancer. National Cancer Institute. Bethesda MD.

[6] Fendler WP, Eiber M, Beheshti M, Bomanji J, Calais J, Ceci F, et al. PSMA PET/CT: joint EANM procedure guideline/SNMMI procedure standard for prostate cancer imaging 2.0. Eur J Nucl Med Mol Imaging. 2023;50:1466–86. 10.1007/s00259-022-06089-w. 36604326 10.1007/s00259-022-06089-w PMC10027805

[7] Sartor O, de Bono J, Chi KN, Fizazi K, Herrmann K, Rahbar K, et al. Lutetium-177-PSMA-617 for Metastatic Castration-Resistant Prostate Cancer. N Engl J Med. 2021;385:1091–103. 10.1056/NEJMoa2107322. 34161051 10.1056/NEJMoa2107322 PMC8446332

[8] Sathekge M, Bruchertseifer F, Vorster M, Lawal IO, Knoesen O, Mahapane J, et al. Predictors of Overall and Disease-Free Survival in Metastatic Castration-Resistant Prostate Cancer Patients Receiving (225)Ac-PSMA-617 Radioligand Therapy. J Nucl Med. 2020;61:62–9. 10.2967/jnumed.119.229229. 31101746 10.2967/jnumed.119.229229

[9] de Galiza Barbosa F, Queiroz MA, Nunes RF, Costa LB, Zaniboni EC, Marin JFG, et al. Nonprostatic diseases on PSMA PET imaging: a spectrum of benign and malignant findings. Cancer Imaging. 2020;20:23. 10.1186/s40644-020-00300-7. 32169115 10.1186/s40644-020-00300-7 PMC7071711

[10] Heitkotter B, Trautmann M, Grunewald I, Bogemann M, Rahbar K, Gevensleben H, et al. Expression of PSMA in tumor neovasculature of high grade sarcomas including synovial sarcoma, rhabdomyosarcoma, undifferentiated sarcoma and MPNST. Oncotarget. 2017;8:4268–76. 10.18632/oncotarget.13994. 28002805 10.18632/oncotarget.13994 PMC5354830

[11] Kleiburg F, Heijmen L, Gelderblom H, Kielbasa SM, Bovee JV, De Geus-Oei LF. Prostate-specific membrane antigen (PSMA) as a potential target for molecular imaging and treatment in bone and soft tissue sarcomas. Br J Radiol. 2023;96:20220886. 10.1259/bjr.20220886. 36728839 10.1259/bjr.20220886 PMC10161918

[12] Militano V, Afaq A, Bomanji J. 68Ga-Prostate-Specific Membrane Antigen PET/CT: Incidental Finding of a Liposarcoma. Clin Nucl Med. 2019;44:e90–2. 10.1097/RLU.0000000000002389. 30608915 10.1097/RLU.0000000000002389

[13] Juptner M, Marx M, Zuhayra M, Lutzen U. Experimental 177Lu-PSMA-617 radioligand therapy in a patient with extended metastasized leiomyosarcoma. Nuklearmedizin. 2019;58:328–30. 10.1055/a-0914-2486. 31140181 10.1055/a-0914-2486

[14] Digklia A, Boughdad S, Homicsko K, Dromain C, Trimech M, Dolcan A, et al. First communication on the efficacy of combined < sup > 177 Lutetium-PSMA with immunotherapy outside prostate cancer. J Immunother Cancer. 2022;10. 10.1136/jitc-2022-005383. 10.1136/jitc-2022-005383 PMC9615971 36288828

[15] Inanir S, Kesim S, Ergelen R, Tinay I, Turkoz HK. 68Ga-PSMA PET/CT in Giant Retroperitoneal Liposarcoma. Clin Nucl Med. 2019;44:e612–3. 10.1097/RLU.0000000000002762. 31524684 10.1097/RLU.0000000000002762

[16] Hohberg M, Kobe C, Krapf P, Tager P, Hammes J, Dietlein F, et al. Biodistribution and radiation dosimetry of [(18)F]-JK-PSMA-7 as a novel prostate-specific membrane antigen-specific ligand for PET/CT imaging of prostate cancer. EJNMMI Res. 2019;9:66. 10.1186/s13550-019-0540-7. 31346821 10.1186/s13550-019-0540-7 PMC6658635

[17] Boellaard R, O’Doherty MJ, Weber WA, Mottaghy FM, Lonsdale MN, Stroobants SG, et al. FDG PET and PET/CT: EANM procedure guidelines for tumour PET imaging: version 1.0. Eur J Nucl Med Mol Imaging. 2010;37:181–200. 10.1007/s00259-009-1297-4. 19915839 10.1007/s00259-009-1297-4 PMC2791475

[18] Soret M, Bacharach SL, Buvat I. Partial-volume effect in PET tumor imaging. J Nucl Med. 2007;48:932–45. 10.2967/jnumed.106.035774. 17504879 10.2967/jnumed.106.035774

[19] Kratochwil C, Fendler WP, Eiber M, Baum R, Bozkurt MF, Czernin J, et al. EANM procedure guidelines for radionuclide therapy with (177)Lu-labelled PSMA-ligands ((177)Lu-PSMA-RLT). Eur J Nucl Med Mol Imaging. 2019;46:2536–44. 10.1007/s00259-019-04485-3. 31440799 10.1007/s00259-019-04485-3

[20] Hofman MS, Violet J, Hicks RJ, Ferdinandus J, Thang SP, Akhurst T, et al. [(177)Lu]-PSMA-617 radionuclide treatment in patients with metastatic castration-resistant prostate cancer (LuPSMA trial): a single-centre, single-arm, phase 2 study. Lancet Oncol. 2018;19:825–33. 10.1016/S1470-2045(18)30198-0. 29752180 10.1016/S1470-2045(18)30198-0

[21] Jansen BHE, Kramer GM, Cysouw MCF, Yaqub MM, de Keizer B, Lavalaye J, et al. Healthy Tissue Uptake of (68)Ga-Prostate-Specific Membrane Antigen, (18)F-DCFPyL, (18)F-Fluoromethylcholine, and (18)F-Dihydrotestosterone. J Nucl Med. 2019;60:1111–7. 10.2967/jnumed.118.222505. 30630941 10.2967/jnumed.118.222505 PMC6910637

[22] Uijen MJM, Derks YHW, Merkx RIJ, Schilham MGM, Roosen J, Prive BM, et al. PSMA radioligand therapy for solid tumors other than prostate cancer: background, opportunities, challenges, and first clinical reports. Eur J Nucl Med Mol Imaging. 2021;48:4350–68. 10.1007/s00259-021-05433-w. 34120192 10.1007/s00259-021-05433-w PMC8566635

[23] Haas RL, Floot BGJ, Scholten AN, van der Graaf WTA, van Houdt W, Schrage Y, et al. Cellular Radiosensitivity of Soft Tissue Sarcoma. Radiat Res. 2021;196:23–30. 10.1667/RADE-20-00226.1. 33914890 10.1667/RADE-20-00226.1

[24] Wang JH, Kiess AP. PSMA-targeted therapy for non-prostate cancers. Front Oncol. 2023;13:1220586. 10.3389/fonc.2023.1220586. 37645427 10.3389/fonc.2023.1220586 PMC10461313

[25] Dietlein F, Hohberg M, Kobe C, Zlatopolskiy BD, Krapf P, Endepols H, et al. An (18)F-Labeled PSMA Ligand for PET/CT of Prostate Cancer: First-in-Humans Observational Study and Clinical Experience with (18)F-JK-PSMA-7 During the First Year of Application. J Nucl Med. 2020;61:202–9. 10.2967/jnumed.119.229542. 31324713 10.2967/jnumed.119.229542

